# LGR5 promotes hepatocellular carcinoma metastasis through inducting epithelial-mesenchymal transition

**DOI:** 10.18632/oncotarget.15143

**Published:** 2017-02-07

**Authors:** Jie Liu, Guo-Zheng Yu, Xiao-Ke Cheng, Xiao-Dong Li, Xian-Tao Zeng, Xue-Qun Ren

**Affiliations:** ^1^ Department of General Surgery, Huaihe Hospital of Henan University, Kaifeng 475000, Henan Province, China; ^2^ Nursing Department, Huaihe Hospital of Henan University, Kaifeng 475000, Henan Province, China; ^3^ Department of General Surgery, Huangshi Central Hospital, Affiliated Hospital of Hubei Polytechnic University, Huangshi 435000, Hubei Province, China; ^4^ Center for Evidence-Based Medicine, Huaihe Hospital of Henan University, Kaifeng 475000, Henan Province, China; ^5^ Department of Urology, Huaihe Hospital of Henan University, Kaifeng 475000, Henan Province, China; ^6^ Center for Evidence-Based and Translation Medicine, Zhongnan Hospital of Wuhan University, Wuhan 430071, Hubei Province, China

**Keywords:** hepatocellular carcinoma, LGR5, leucine-rich repeat-containing G-protein coupled receptor 5, metastasis, epithelial-mesenchymal transition

## Abstract

The purpose of the present study was to investigate the prognostic value of Leucine-rich repeat-containing G-protein coupled receptor 5 (LGR5) in hepatocellular carcinoma (HCC) and its role in promoting HCC metastasis. The expression level of LGR5 in liver tumor tissues and adjacent non-tumor tissues were detected adopting immunohistochemistry (IHC), real-time PCR (RT-PCR) and western blot assays. Chi-square test was used to evaluate the correlation between LGR5 expression and clinicopathological characteristics. In addition, we assessed the relationship between LGR5 and two epithelial-mesenchymal transition (EMT) markers (E-cadherin and N-cadherin) in HCC tissues and cell lines. Our results showed that the expression of LGR5 was significantly higher in liver tumor tissues than in adjacent non-tumor tissues. Moreover, up-regulated LGR5 was associated with larger tumor diameter (>5cm, P=0.001), higher TNM stage (P=0.021), increased recurrence (P=0.023) and growing metastasis (P=0.030). Besides, we found that the expression level of LGR5 was correlated with E-cadherin and N-cadherin. In conclusion, up-regulated LGR5 in HCC patients is associated with malignant clinicopathological characteristics. LGR5 may promote HCC metastasis through inducting EMT process, and thus can be regarded as a candidate biomarker for prognosis and as a target in therapy.

## INTRODUCTION

Hepatocellular carcinoma (HCC) is one of the most common malignancies in the world, and no effective drug intervention has been developed yet for its treatment [1–2]. Annually, HCC sees over one million new cases worldwide, representing the 5th most common cancer and the 3rd leading cause of cancer-related death (preceded only by lung cancer and gastric cancer) [3–7]. Recently, several proteins and signaling pathways have been found to be correlated with the prognosis of HCC. The mechanisms of metastasis and recurrence in HCC patients have not been thoroughly understood, so explorations of prognostic molecular markers for this malignancy are still essential.

Leucine-rich repeat-containing G-protein coupled receptor 5 (LGR5), also known as G-protein coupled receptor 49 (GPR49) or G-protein coupled receptor 67 (GPR67), is a protein encoded by the LGR5 gene in humans [8]. LGR5 is expressed by a diverse range of tissues and organs, such as muscle, placenta, spinal cord and brain, and particularly acts as a biomarker for adult stem cells in certain tissues [9].

LGR5 is crucial for tumor development and tumor cell signal transduction. In this study, we explored whether or not LGR5 was involved in the tumor development and cell signal transduction in HCC. We measured the expression levels of LGR5, E-cadherin, and N-cadherin in HCC tissue and HCC cell lines using immunohistochemical, western blot and *in vitro* experiments to explore the relationship between LGR5 and epithelial-mesenchymal transition (EMT) in HCC. The results from this study could serve as theoretical and experimental bases for HCC diagnosis, treatment, and prevention.

## RESULTS

### LGR5, N-cadherin and E-cadherin mRNA and protein expression in HCC tissue

**Figure 1:**
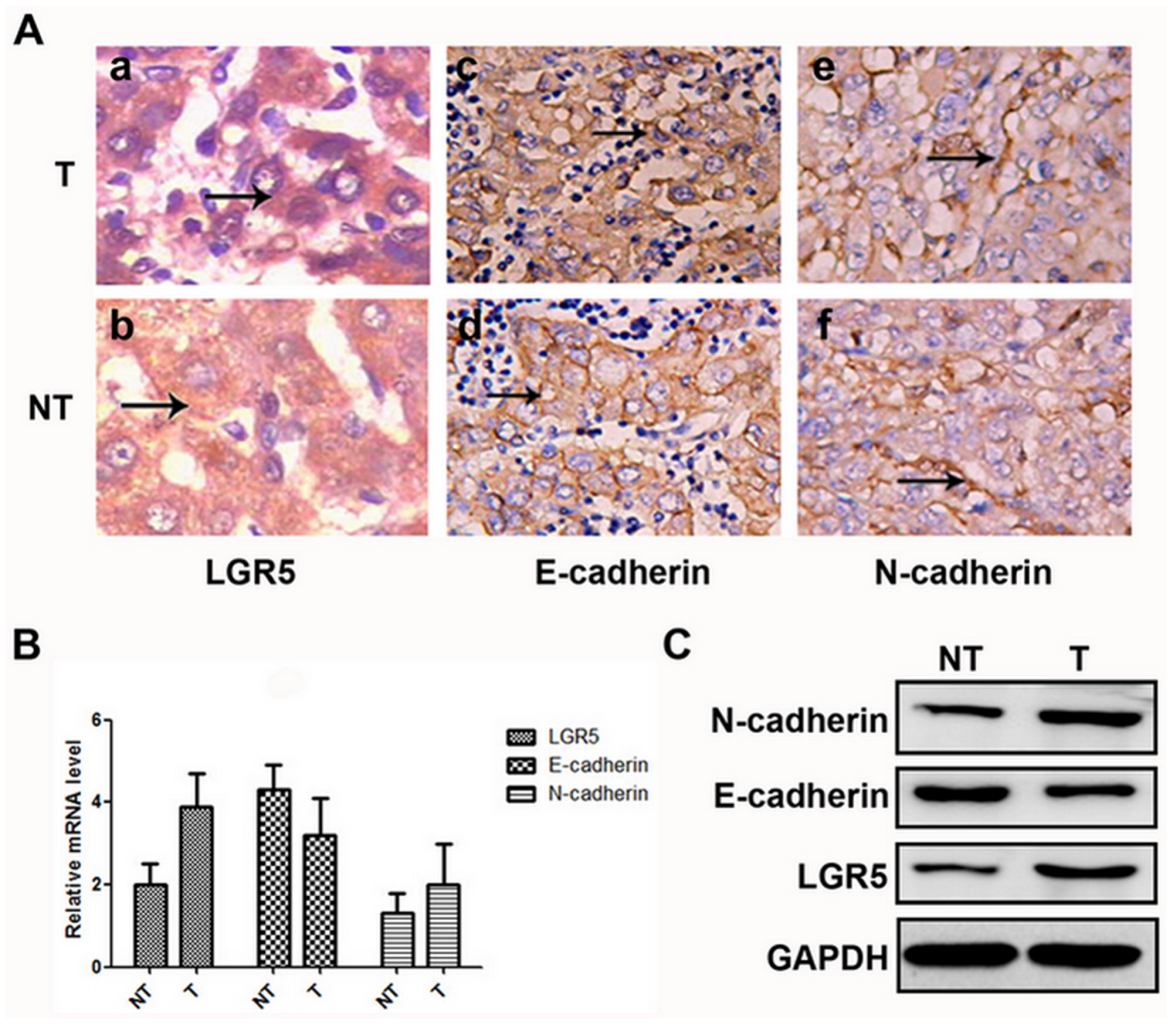
Expressions of LGR5, E-cadherin, and N-cadhe in HCC samples **A.** Hematoxylin and eosin staining through morphologic changes. These cells express high levels of LGR5 (a and b) and N-cadherin (e and f), but low levels of E-cadherin (c and d). **B.** Relative mRNA expressions of LGR5, E-cadherin and N-cadherin in tumor and adjacent non-tumor tissues, respectively. **C.** Western blot analysis of LGR5, E-cadherin and N-cadherin in tumor and adjacent non-tumor tissues, respectively.

In 139 pairs of tissues, 88.5% (123/139) of HCC tumor tissues showed positive expression of LGR5, so did 11.5% (13/139) of the adjacent non-tumor tissues. The positive rate of LGR5 protein was significantly higher in HCC tissues than in adjacent non-tumor ones (88.5% vs. 11.5%, P<0.05). In addition, 27.3% (38/139) of tumor tissues showed high expression of E-cadherin, while such ratio was 72.7% (101/139) in adjacent non-tumor tissues. The positive rate of E-cadherin protein was significantly lower in HCC than in adjacent non-tumor tissues (27.3% vs. 72.7%, P<0.05), and 66.9% (93/139) of the tumor tissues and 33.1% (46/139) of the adjacent non-tumor tissues showed high expression of N-cadherin. The positive rate of N-cadherin protein was significantly higher in HCC tissues than in adjacent non-tumor ones (66.9% vs. 33.1%, P < 0.05) (Figure 1a-1f).

The mRNA expression of LGR5, N-cadherin and E-cadherin in HCC tissues were also compared with those in corresponding adjacent non-tumor tissues using real-time PCR. As a result, LGR5 and N-cadherin mRNA expression were significantly higher in HCC tissues than in those non-tumor tissues. Whereas, E-cadherin just showed an opposite tendency (Figure 1B). Consistent results were also detected by Western blot (Figure 1C).

### Overexpression of LGR5 is associated with EMT in HCC cell lines

**Figure 2:**
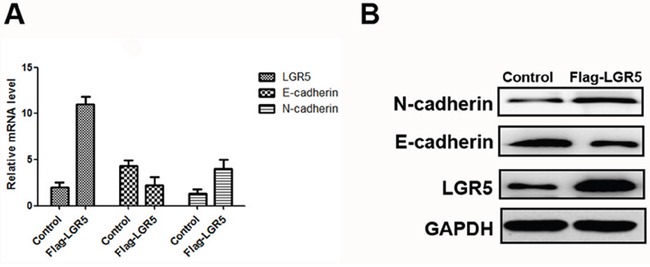
Expressions of LGR5, E-cadherin, and N-cadherin in Huh7 cell lines The transfection of Flag-LGR5 plasmid into Huh7 cells. **A.** Relative mRNA expressions of LGR5, E-cadherin and N-cadherin in Huh7 cell lines. **B.** Western blot analysis of LGR5, E-cadherin and N-cadherin in Huh7 cells.

RT-PCR and Western blot assays were applied to detect relative expression levels of LGR5, N-cadherin and E-cadherin in transfected cells. The results indicated that, the expression level of E-cadherin was significantly lower in LGR5-overexpression group than in control group. But the expression level of N-cadherin was significantly higher in LGR5-overexpression than in control group (Figure 2a-2b). Therefore, these two factors were key markers for tumor metastasis, indicating that LGR5 took part in EMT process [10]. These results demonstrated that LGR5 might play an important role in promoting EMT process.

### Disturbance of LGR5 is associated with EMT in HCC cell lines

**Figure 3:**
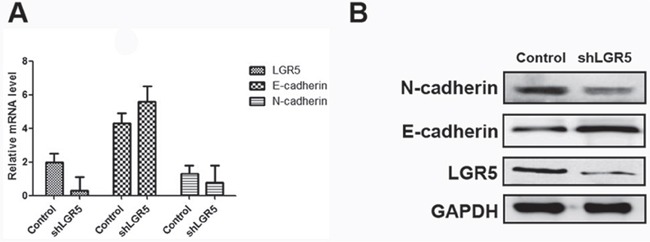
Expressions of LGR5, E-cadherin, and N-cadherin in Huh7 cell lines The transfection of shLGR5 plasmid into Huh7 cells with inhibited LGR5 expression. **A.** Relative mRNA expressions of LGR5, E-cadherin and N-cadherin in Huh7 cell lines. **B.** Western blot analysis of LGR5, E-cadherin and N-cadherin in Huh7 cells.

To further examine the relationship between LGR5 and the EMT process, we investigated the changes in EMT markers between LGR5-shRNA and control group using real time-PCR and Western blot. Both analyses revealed that HCC cells with inhibited LGR5 expression had down-regulated N-cadherin and up-regulated E-cadherin (Figure 3a-3b).

### MTS assay for analysis of Huh7 cells

**Figure 4:**
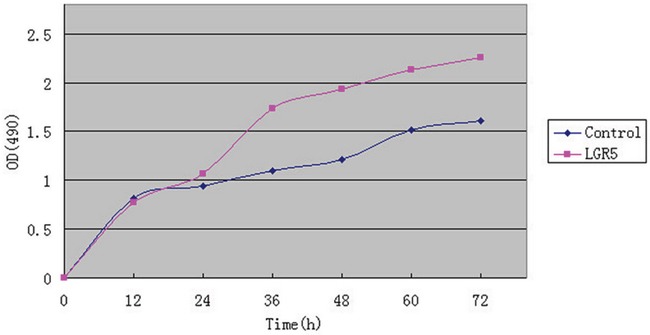
MTS assay for analysis of Huh7 cells The transfection of Flag-LGR5 plasmid into Huh7 cells.12 and 24 hours after transfection the OD values was not statistically significant in either Flag-LGR5 plasmid group or control group. 36, 48, 60, and 72 hours after transfection, the OD values were significantly higher in the both groups (P < 0.05). Results indicated that LGR5 could promote Huh7 cells proliferation.

Huh7 cells were transfected with plasmid Flag-LGR5 and control Flag-2b. 12 and 24 hours after transfection, the OD values was not statistically significant in Flag-LGR5 plasmid group or in control group. But 36, 48, 60, and 72 hours after transfection, the OD values was significantly higher in these two groups (P<0.05, Figure 4). Results indicated that LGR5 could promote Huh7 cells proliferation.

### Relationships between the expressions of LGR5, E-cadherin and N-cadherin and clinicopathological features of HCC patients

The correlation between these proteins and clinicopathological characteristics of HCC is summarized in Table 1. Up-regulated expression of LGR5 was significantly correlated with larger tumor diameter (>5cm, P=0.001), higher TNM stage (P=0.021), recurrence (P=0.023) and metastasis (P=0.030) in HCC. Moreover, the expression of N-cadherin and E-cadherin were both significantly associated with tumor diameter, TNM stage, recurrence and metastasis of HCC as well (Table 1).

**Table 1:** Correlations of LGR5, E-cadherin and N-cadherin staining with clinicopathological factors in 139 HCC patients

Variables	LGR5		*P*	E-cadherin		*P*	N-cadherin		*P*
	High	Low		High	Low		High	Low	
**Age(years)**									
≤ 50	75	9	0.716	22	64	0.554	55	28	0.77
> 50	48	7		16	37		38	18	
**Gender**									
Male	99	13	1	31	81	0.542	74	38	0.67
Female	24	3		7	20		19	8	
**Tumor diameter (cm)**									
>5	85	3	0.001	13	32	0.017	57	15	0.001
≤5	38	13		25	69		36	31	
**TNM stage**									
I	34	9	0.021	6	37	0.013	30	13	0.032
II	51	5		13	43		35	11	
III	33	2		17	18		24	11	
IV	5	0		2	3		4	1	
**Grade**									
I	30	4	0.957	7	27	0.31	18	16	0.046
II	93	12		31	74		75	30	
**HBsAg**									
Yes	117	13	0.069	34	96	0.257	86	44	0.718
No	6	3		4	5		7	2	
**Liver cirrhosis**									
Yes	108	12	0.236	32	88	0.655	79	41	0.499
No	15	4		6	13		14	5	
**Liver function**									
A	69	13	0.247	24	58	0.315	55	27	0.426
B	54	3		14	43		38	19	
C	0	0		0	0		0	0	
**AFP(ng/ml)**									
>400	47	7	0.669	13	41	0.491	38	16	0.489
≤ 400	76	9		25	60		55	30	
**Recurrence(2years)**									
Yes	105	10	0.023	24	85	0.007	84	33	0.005
No	18	6		14	16		9	13	
**Metastasis(≤24 month)**									
Yes	87	7	0.03	20	74	0.02	70	24	0.006
No	36	9		18	27		23	22	

AFP: a-fetoprotein; HBsAg: hepatitis B virus surface antigen; TNM: tumor-node-metastasis.

## DISCUSSION

The present study showed that the expression of LGR5 was markedly higher in HCC than in normal liver tissues. Up-regulated expression of LGR5 was significantly correlated with larger tumor diameter (>5cm, P=0.001), higher TNM stage (P=0.021), recurrence (P=0.023) and metastasis (P=0.030) in the malignancy. These findings strongly commended LGR5 as a predictor for tumor aggressiveness and prognosis in HCC. Furthermore, we also found that the level of LGR5 expression was significantly correlated with two epithelial-mesenchymal transition (EMT) markers. The shRNA disturbed LGR5 expression in HCC cell line, thereby resulting in downregulation of N-cadherin and upregulation of E-cadherin. According to our findings, LGR5 might promote HCC metastasis through inducting EMT process, and thus could be used as a candidate biomarker for prognosis as well as a target for the cancer therapy.

HCC invasion and metastasis involve decreased adhesion between tumor cells, damages in the extracellular matrix structure, movement of tumor cells, and formation of new blood vessels in tumors. In adhesion, HCC cells extend their pseudopodia to surrounding tissues and adhere to extracellular matrix. In degradation, HCC cells can induce the expression of various factors and then produce a substantial amount of hydrolytic enzymes which can degrade extracellular matrix and damage basement membrane, thereby allowing tumor cells to escape. Tumor cells moving out from the basement membrane can travel great distances. The formation of new blood vessels is necessary for proliferation and metastasis of HCC cells. EMT links the invasion and metastasis of tumor cells. During EMT, epithelial phenotype transforms into mesenchymal phenotype [11]. In addition, since epithelium loses cell polarity, intercellular connection disappears, and cytoskeleton is restructured, the function of intercellular adhesion vanishes. EMT appearance evolves into the spindle fiber cell morphology and transforms into mesenchymal phenotype [12]. The enhancement of adhesion between cells and stromal cells enforces the migration and movement capability of tumor cells. At the same time, cell phenotype down-regulates the expression of E-CAD while up-regulates the expression of N-CAD. Using RT-PCR, Yamamoto et al.[13] found that LGR5 mRNA was overexpressed in HCC. β-catenin is the key effector in Wnt/β-catenin signaling pathway, and the third exon of β-catenin gene segment is mutated frequently. This fact indicates that LGR5 may be associated with β-catenin and thus induce EMT through the Wnt/β-catenin signaling pathway. In this signaling pathway, Fizzled can act on the DVL at the cytoplasm. After Wnt combines with Fizzled, the protein complex colonic adenomatous polyposis coli (APC)/shaft protein (axin)/3b glycogen synthase kinase (GSK-3b) loses stability; the phosphorylation level of β-catenin decreases, and a large number of β-catenin congregate on the cytoplasm; and β-catenin combines with the member of T cell factor/lymphoid enhancer factor (TCF/LEF), thus regulating the expression of target genes of the Wnt signaling pathway. Meanwhile, the Wnt/β-catenin signaling pathway is also important for EMT cells [14–15]. Zhao et al. found that this pathway might be directly controlled by hypoxia-inducible factor-1α to induce EMT [16].

The mechanisms of the functional roles of LGR5 in enhancing different aspects of tumor malignancy remain poorly defined. One novel finding in this study is that LGR5 expression was closely associated with key markers of EMT, such as E-cadherin, and N-cadherin. EMT is a process during which cells change from cobble-stone shapes that exhibit tight cell-cell contact into spindle-shaped fibroblast-like shapes that lose cell-cell contact and cell polarity [17]. In tumor cells, this process can result in an increase in mesenchymal-like cells and a decrease in epithelial-like cells, thus enhancing the invasive and metastatic capacity of tumors. Because EMT also plays an important role in HCC invasion and metastasis [18], a positive correlation between LGR5 expression and EMT process may provide an explanation for the role of LGR5 in HCC malignancy.

In conclusion, this study shows that LGR5 promotes HCC metastasis through inducting EMT process. The evidence presented in this study strongly suggests that LGR5 can be considered as a candidate biomarker for HCC prognosis and as a target in the malignancy therapy.

## MATERIALS AND METHODS

### Cell line culture

Human HCC cell-line Huh7 was obtained from the Cell Bank of the Chinese Academy of Sciences (Shanghai, China). All cells were cultured in the recommended media supplemented with 10% (v/v) fetal bovine serum at 37°C in an incubator with 5% CO2.

### Patients and specimens

Samples from 139 Chinese HCC patients were used in this study. These patients underwent curative liver resection for primary tumors between March 2013 and November 2015 at Huaihe Hospital of Henan University. They had received no chemotherapy or radiation therapy before surgery. Both tumor tissues and corresponding adjacent non-tumor tissues were selected for each patient. Adjacent non-tumor tissues were at least 2 cm from the edge of the tumor. All of the patients were pathologically diagnosed with HCC. The clinicopathological features of included patients were abstracted from medical charts using a standard protocol. The tumors were graded using the Nottingham combined histologic grading system, and TNM staging was implemented according to the American Joint Committee on Cancer (AJCC) recommendations. Of the 139 HCC patients, 112 (80.6%) were males and 27 (19.4%) were females, with a median age of 51.7 years. The follow-up time ranged from 1 to 60 months with a median duration of 31.15 months. The detailed clinicopathological characteristics of the patients are listed in Table 1. This study was approved by the research ethics committee of Huaihe Hospital of Henan University, and informed consent was obtained from each patient. We performed all experiments in accordance with relevant guidelines and regulations.

### Immunohistochemistry (IHC)

Both HCC tissues and adjacent non-tumor liver tissues were sectioned with a 4-mm thickness. The tissue samples were immersed in buffered formalin and embedded in paraffin following standard procedures. The tissue sections were deparaffinized in xylene and rehydrated using graded ethanol, and endogenous peroxidase was inactivated with 0.3% hydrogen peroxide for 10 mins. Then, the tubes were tilted to allow serum to mix with 50 μL of diluted antibody (E-cadherin, N-cadherin, and LGR5 antibodies; dilution ratio = 1:150). The sections were placed in refrigerator at 4 °C overnight. Subsequently, the sections were added with biotin labeling antibody (50 μL), incubated at 37 °C for 15 mins, added with streptomycete anti-biotin peroxidase (50 μL) dropwise, and then incubated again at 37 °C for 15 mins. Next, the sections were immersed in chromogenic reagent diaminobenzidine for 3 mins, stained with hematoxylin dye for 3 mins, and then submerged in 1% hydrochloric acid and ethanol solution for 30 s. Finally, the sections were further processed for dehydration and transparence, and then sealed with neutral gum. A negative control group and a positive control group were also established. PBS solution instead of the first antibody was used in the negative control group, and the known antigen was utilized in the positive control group.

### IHC evaluation

The total immunostaining score was calculated as the sum of the positive percentage and staining intensity of the stained cells. Two doctors in our hospital were randomly assigned to count the cells in a double-blind manner. Each slice was randomly selected and observed under high magnification (400×). Each field had 100 cells, and the percentage of positive cells in each section was calculated. The score standard for staining intensity was based on different degrees of tissue staining without specific background staining: no visible staining, 0; light yellow, 1; tan, 2; and dun, 3. The score standard for positive cell evaluation criteria was as follows: positive cells <5%, 0; >5% but <25%, 1; >25% but <50%, 2; >50% but <75%, 3; and >75%, 4. The total score was obtained through multiplying staining power by positive cell percentage. A total score exceeding 4 indicated positive expression, whereas a total score below 4 indicated negative expression.

### Real-time PCR and western blot

Total RNA was isolated using Trizol reagent (15596-026; Invitrogen) and reversely transcribed to cDNA with PrimeScript RT reagent kit (DRR037A; Takara). For real-time PCR (RT-PCR), SYBR Premix Ex Taq (DRR081; Takara) was used according to the manufacturer's instructions. In Western blot analysis, RIPA lysis buffer was used to determine total protein concentration with Bradford assay. Up to 100 μg of total protein was processed with 10% SDS-PAGE gel and then transferred onto nitrocellulose membranes. The membranes were blocked in 5% blocking solution for 2 hrs and then incubated overnight with primary rabbit anti-human polyclonal antibodies (1:2000) at 4 °C. The membranes were washed and then incubated with secondary goat anti-rabbit IgG-HRP antibodies (1:5000) for 1h. The membranes were washed after the film ECL color., and the images were captured applying Gel Doc XR system (Bio-Rad) and analyzed employing Image Lab soft-ware (version 2.0). The primers in this study are listed in Table 2.

**Table 2:** Primer Sequence for real-time PCR

Protein	Name	Sequence *(5′→3′)*
LGR5	F	AGGTCAGGTGAAGCGCTCG
	R	CGTTGCAACACTGTCATGGC
E-cadherin	F	ACCGGGACAACGTTTATTACTATGA
	R	TAAGCCGCTTACATTCGGTTA
N-cadherin	F	CAGGTTCGCATGCCCAAGCATC
	R	ATGCCCGTAACGTCGCCACGT

### Cell transfection

The functional role of LGR5 in HCC cells was assessed via transfecting short hairpin RNA (shRNA) and LGR5 plasmid. Huh7 cells were cultured in logarithmic phase and then experienced trypsin digestion. Cell concentration was adjusted in each well (inoculated with 2 ml) of the six-well plates. When cell concentration reached approximately 70%, cells were transfected with corresponding vectors using Lipofectamine 2000 (Invitrogen) following the manufacturer's protocol. The samples were incubated for 48 h following the above mentioned procedures, and then subjected to Real-time -PCR and Western blot analyses. Experiments were repeated thrice.

### Cell viability assay (MTS assay)

Huh7 cells (5 × 10^3^ cells per well) were plated in 96-well plates in 100 mL media. According to the Lipo 2000 manual steps, cells were transfected with plasmid Flag-LGR5 and control Flag-2b. Cell viability was quantitated via MTS colorimetric techniques using the Cell Titer 96 Aqueous One Solution Cell Proliferation Assay. After 12, 24, 36, 48, 60 and 72 hours, the optical density (OD) values were detected separately at 490nm wavelength. Each experiment was performed at least in triplicate.

### Statistical analysis

Chi-square test was used to evaluate the correlation between LGR5 and clinicopathological characteristics. The levels of mRNA expressions were analyzed by student's t-test, and repeated measure data was analyzed using ANOVA. Patients alive at the end of follow-up were censored. The recurrence time was defined as a period from HCC resection to the first day obtaining radiological evidence for the event. Statistical analysis was performed using SPSS ver. 18.0 for Windows (SPSS, Chicago, IL, USA).
